# Improving health literacy using the power of digital communications to achieve better health outcomes for patients and practitioners

**DOI:** 10.3389/fdgth.2023.1264780

**Published:** 2023-11-17

**Authors:** Patrick J. Fitzpatrick

**Affiliations:** Educate4Health, Dublin, Ireland

**Keywords:** health literacy (HL), digital communications in healthcare, clinical decision support systems (CDSS), AI in healthcare, health inequities and inclusion, digital health regulation, digital health governance

## Abstract

Digital communication tools have demonstrated significant potential to improve health literacy which ultimately leads to better health outcomes. In this article, we examine the power of digital communication tools such as mobile health apps, telemedicine and online health information resources to promote health and digital literacy. We outline evidence that digital tools facilitate patient education, self-management and empowerment possibilities. In addition, digital technology is optimising the potential for improved clinical decision-making, treatment options and communication among providers. We also explore the challenges and limitations associated with digital health literacy, including issues related to access, reliability and privacy. We propose leveraging digital communication tools is key to optimising engagement to enhance health literacy across demographics leading to transformation of healthcare delivery and driving better outcomes for all.

## Introduction

**Figure 1:**
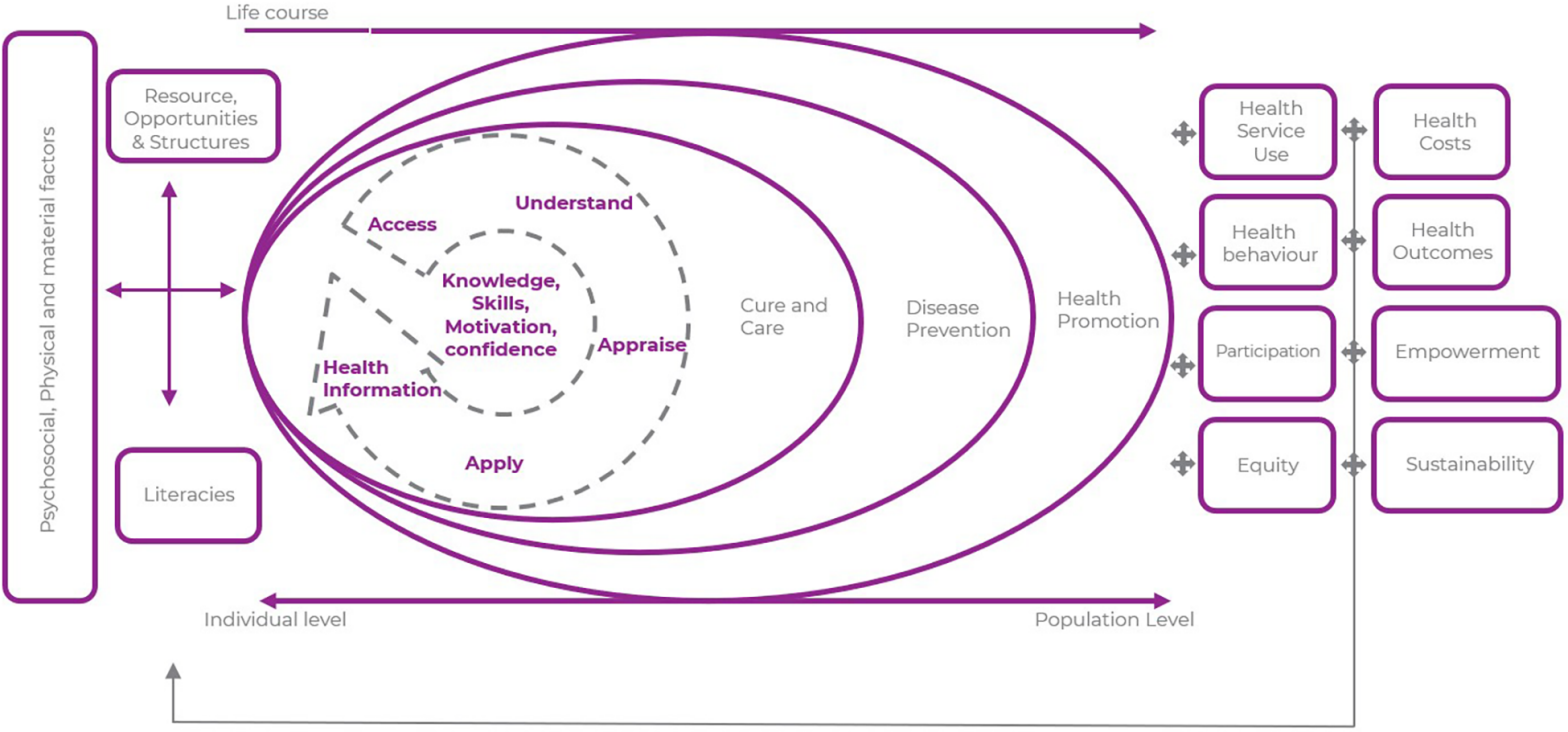
Illustration of the various dimensions of health literacy and the impact on different health domains. [Adapted from HLS-EU 2019 (13)].

Health literacy, illustrated in Figure 1, is defined as the ability to obtain, comprehend and apply health information to make informed decisions related to health (1, 2)_,_ has been demonstrated to help improve health status (3, 4). Digital literacy is described as an individual's ability to find, evaluate and communicate information by using digital tools (5). Health and digital literacy play a vital role in promoting better health outcomes for individuals and communities (3, 4, 6). Limited health literacy has been linked to poor health outcomes, increased healthcare costs and health disparities (7, 8). To address these issues there has been growing recognition of the potential of digital communication tools to improve health literacy and empower individuals to take a more active role in managing their health (9–12).

Digital communication tools, such as mobile health apps, telemedicine and online health information resources, have gained significant popularity and are increasingly being integrated into healthcare delivery systems. These tools offer unique opportunities to reach a wide range of demographics, regardless of their geographic location, socioeconomic status, or educational background. By leveraging the power of technology, digital communication tools have the potential to enhance health literacy, improve patient-provider communication and ultimately lead to better health outcomes.

This review aims to explore the power of digital communication tools in improving health literacy and achieving better health outcomes for both patients and practitioners. We examine the current evidence on the effectiveness of various digital tools, including mobile health apps, telemedicine platforms and online health information resources, in promoting health literacy. The impacts of improved health literacy, the challenges associated with low digital health literacy and strategies to overcome these constraints are discussed. We also assess the implications of leveraging digital communication tools for optimising engagement, enhancing health literacy across different demographics and transforming healthcare delivery.

**Figure 2:**
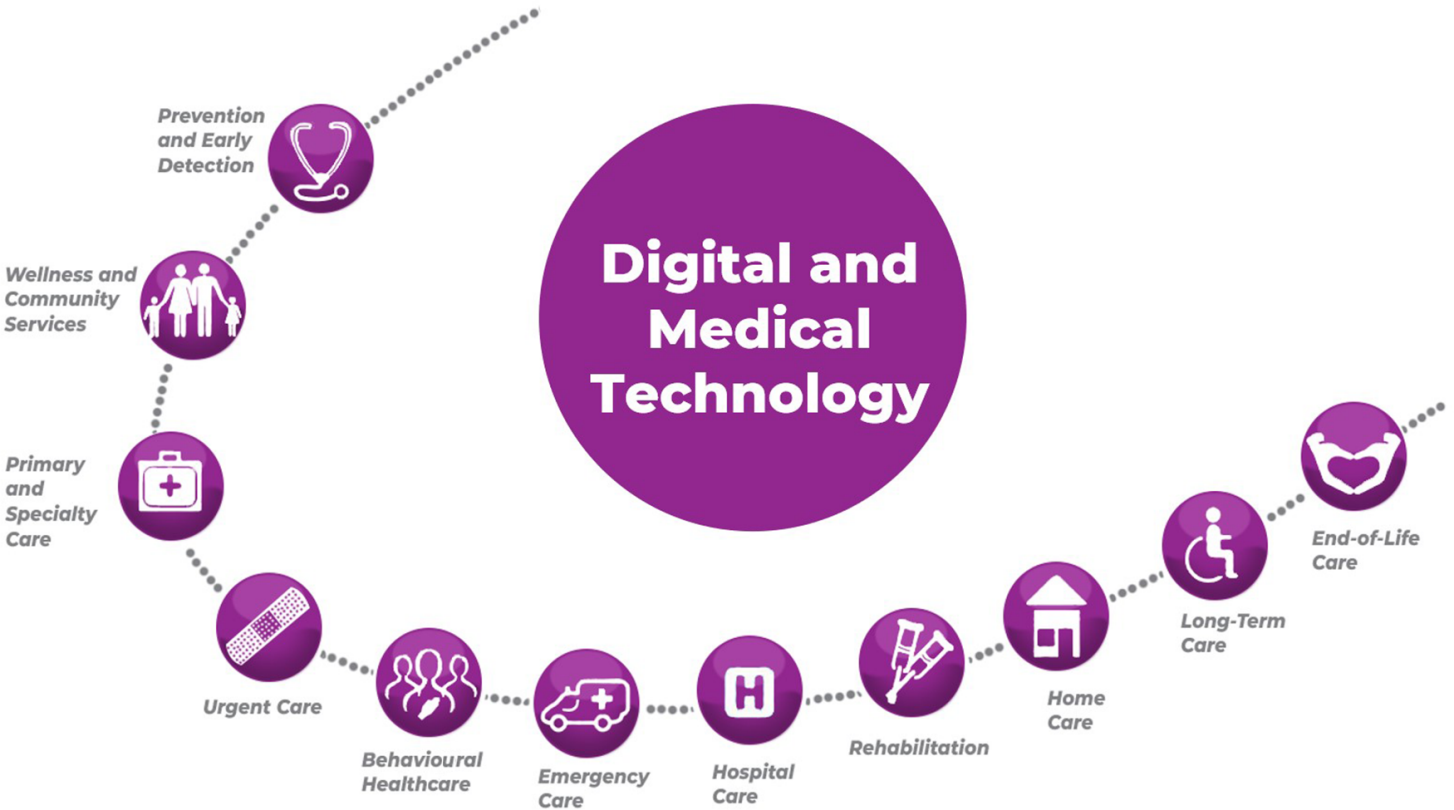
Full spectrum of healthcare services.

We aim to provide a deeper understanding of the potential of digital communication tools to improve health literacy and contribute to better health outcomes across the full spectrum of healthcare (Figure 2). By identifying the benefits, limitations and future directions of digital health literacy interventions, this review will inform researchers, healthcare professionals, policymakers and other stakeholders in effectively utilising digital tools to promote health literacy and deliver patient-centred care.

## Limited health literacy: a global problem

Health literacy levels vary significantly across the world. Poor health literacy remains a significant challenge globally. The consequences of low health literacy are far-reaching, impacting individuals' ability to access, comprehend and act upon health information effectively. The disparities in health literacy levels across countries and regions, underscores the need for targeted interventions to address these disparities.

A population survey conducted by HLS19 Consortium of the WHO Action Network M-POHL (2021) (13) examined health literacy levels in Europe and found considerable variations among countries. The Health Literacy categorical levels of “inadequate” and “problematic” were combined and defined as “limited” Health Literacy. The survey revealed a range for “limited” health literacy of 25%–72%. That means between one in four and three out of four residents in countries participating in HLS19 have limited general Health Literacy.

Šulinskaitė et al. (14) examined health literacy in a cross-sectional survey conducted in Lithuania which showed 40.6% of respondents had problematic health literacy. Inadequate or problematic health literacy was noted among 83.6% of respondents aged 59 years and older. Similar rates were also observed among patients with basic or primary education (76.1%), secondary education (76.6%) and divorced patients (86%). Respondents with better health literacy also had better health behaviours (*p* < 0.05). This study shows the influence of age, education and family status on health literacy.

A review of previous studies in the United States (US) revealed that at least 88% of adults living in the US have health literacy inadequate to navigate the healthcare system and promote their well-being (15). Only 12% are proficiently health literate. Adults with lower health literacy are more likely to return incomplete medical forms/assessment tools, miss appointments with health providers and neglect follow-ups to required medical procedures.

According to the Australian Bureau of Statistics 60% of Australian adults have low Health Literacy (16, 17). A nationally representative study in Canada of men's health literacy showed low income, low education and living alone were associated with men's low Health Literacy (18).

Analysis of Health Literacy studies conducted in South East Asia also found that limited health literacy varied considerably, 1.6%−99.5% with a mean of 55.3%. As with other studies the most common factors associated with limited Health Literacy were education attainment, age, income and socio-economic background (19).

Addressing the global impact of poor health literacy requires tailored interventions that consider the unique challenges faced by different populations (8, 20). Efforts should focus on improving health communication practices, developing culturally appropriate health information materials and strengthening healthcare systems to better meet the needs of individuals with varying health literacy levels (21, 22).

Limited health literacy poses significant challenges to individuals and healthcare systems (8, 23, 24). The cited references shed light on the disparities in health literacy and emphasise the need for targeted interventions to address these gaps (25, 26). By implementing evidence-based strategies and prioritising health literacy as a global agenda, we can empower individuals, reduce health disparities and improve health outcomes for populations worldwide.

## Improving health literacy

Vital to improving health literacy is conveying information in a manner and language that is easily understood and therefore engages the target audiences. It is essential to ensure that health information is accessible, comprehensible and actionable for individuals with varying levels of health literacy. Engagement of any target groups requires presenting the correct information to the relevant people in a timely manner using the most appropriate media. To achieve these objectives effective efficient content creation is fundamental. Producing engaging content that resonates, requires understanding the key messages to be conveyed, identifying the target audiences and their communication preferences.

There are many well established methodologies to help draft content and assess the understandability of language and terminology to convey the relevant health information. One example the Clear Communication initiative promotes the use of plain language in health communication to enhance understandability (27). This approach involves using clear, concise and jargon-free language, avoiding complex medical terminology, providing explanations and examples to improve comprehension (28, 29). In June this year the International Organisation for Standardisation (ISO) formally approved its first international plain language standard (30). The new standard will help improve written communication for everyone.

Readability Assessment Tools, see Table 1 below lists some of the easily accessible resources for healthcare professionals and content creators to ensure the understandability of healthcare information. By actively utilising these freely available assessment tools, healthcare organisations can and should gauge the readability of their materials to enable informed decisions regarding the effectiveness of health information communication. Raising awareness about the availability of these tools and promoting their use can contribute to the development of health content that is clear, concise and tailored to individuals with varying levels of health literacy. Such a proactive approach ensures that healthcare information is comprehensible to the intended audience increasing the probability of engagement and motivating individuals to make informed decisions about their health.

**Table 1:** Some of the well known health communication readability assessment tools.

Readability assessment tool	Description
Automated Readability Index (ARI) (31)	Calculates the readability of a text based on sentence length and word complexity.
Coleman–Liau Index (32)	Evaluates the readability of a text by considering the average sentence length and character count.
Dale–Chall Readability Formula (33)	Assesses the readability of a text based on a list of familiar words and sentence length.
Flesch Reading Ease Score (34)	Provides a measure of the ease of reading a text based on sentence and word length.
FORCAST (Formula for Assessing Readability and Complexity of Text) (35)	Evaluates the readability and complexity of a text based on sentence structure and vocabulary.
Fry Graph Readability Formula (36)	Determines the readability of a text by analysing the number of sentences and syllables per hundred words.
Gunning Fog Index (37)	Determines the readability of a text by analysing sentence length and word complexity.
Simple Measure of Gobbledygook (SMOG) (38)	Estimates the reading grade level of a text by analysing sentence length and polysyllabic word count.

## Overview of digital communication tools

Digital communication tools, including mobile health apps, telemedicine and online health information resources, have emerged as powerful platforms for promoting and improving health literacy leading to better health outcomes. Mobile health apps provide convenient access to health information, self-monitoring tools and personalised interventions, empowering individuals to actively participate in their healthcare journey (39). Telemedicine enables remote consultations, expanding access to healthcare services and facilitating patient-provider communication, particularly in underserved areas (40). Online health information resources offer a wealth of information that can support patient education, decision-making and self-management (41, 42).

However, the so called “digital divide” persists. The digital divide refers to the gap in access to digital technologies and the internet across certain populations and regions, many having limited or no access (43). Such limited access to digital technologies hinders availability and participation in digital activities. Approximately two thirds of the world's population have internet access but there are vast differences in internet usage between high (91%) and low (22%) income countries (44). Developing countries face infrastructure challenges, including lack of connectivity, electricity and access to necessary devices, hindering digital access for a significant portion of their populations. In addition, there are imbalances in terms of digital skills and literacy. Even when individuals have access to digital tools, there may be a lack of proficiency in using them effectively to access information and services online. The gap in digital skills further exacerbates the inequalities in digital access and inhibits the full potential of digital technologies to reach all populations.

Despite the challenges digital access and connectivity continue to improve worldwide (44). Increased investment in infrastructure, expansion of mobile networks and greater affordability of digital technologies are reducing the disparities in digital access (44, 45). The widespread availability of mobile devices and the increasing use of mobile health apps and telemedicine platforms have shown promising results in empowering individuals to actively manage their health and make informed decisions (46). Furthermore, the use of digital technologies enables the delivery of tailored health information and interventions, reaching underserved populations and bridging the gaps in health literacy (47, 48). As digital access continues to expand and technologies become more accessible, the potential for digital communication tools to improve global health literacy continues to increase in significance.

## Promoting health literacy through mobile health apps

Mobile health apps offer various features, such as interactive educational content, symptom tracking, medication reminders and peer support communities, which can enhance health literacy (39). There are over 300,000 health related apps available globally (49). Studies have shown that mobile health apps can improve knowledge and self-efficacy in managing chronic conditions (50). For example, a randomised controlled trial by Greenwell et al. (51) demonstrated that a mobile app for asthma self-management significantly improved patients' knowledge and confidence in using their inhalers. Mobile Health (mHealth) can help achieve global health service coverage by overcoming geographical barriers increasing access for patients in remote areas and communities with insufficient healthcare services (47).

Standards and objective assessment of mobile health apps are increasingly recognised as critical in ensuring app quality, safety, and effectiveness. App certification organisations, such as the Organisation for the Review of Care and Health Apps (ORCHA) (52), play a pivotal role in evaluating and rating health apps based on stringent criteria. ORCHA assesses apps across multiple domains, including clinical effectiveness, data privacy, usability and accessibility, providing users with reliable information to make informed choices (53). Another example is the mHealth App Certification Program by the UK National Health Service (NHS) Digital (54), which evaluates apps against defined technical, clinical and regulatory standards. Similarly, the Health App Certification Program by Happtique (55) provides a certification process that evaluates apps. Objective assessments and certifications enhance user trust and confidence by promoting app transparency, reliability, and adherence to best practices. Objective certification also facilitates healthcare professionals' integration of apps into clinical workflows, ensuring the use of evidence-based and safe digital health tools (56). By establishing standards and offering objective assessments, certification organisations contribute to improving the overall quality and reliability of mobile health apps.

## Enhancing health literacy via telemedicine

Telemedicine platforms enable remote consultations, enabling patients to access healthcare services from their homes. This approach can enhance health literacy by providing real-time interactions with healthcare providers, fostering patient education and facilitating shared decision-making (40). Research has shown that telemedicine consultations can improve patient satisfaction, reduce travel-related barriers resulting in increased patient knowledge and understanding of their conditions (57, 58).

## Online health information resources and health literacy

The availability of online health information resources has transformed how individuals seek and obtain health-related information. These resources, including reputable websites, online forums and social media platforms, offer opportunities for patients to access a wide range of health information and support (50, 59–61). However, the reliability and accuracy of online information can vary dramatically. Individuals with low health literacy may struggle to navigate and comprehend complex health content (62). Therefore, efforts to ensure the quality and accessibility of online health information are crucial to improving health literacy. Table 2 provides examples of how various different digital tools have been used to improve health literacy.

**Table 2:** Examples of digital tools' impact on health literacy.

Reference	Digital tool	Impact on health literacy
Barello et al. (63)	Mobile health apps	Demonstrated success in providing personalised education and support to patients, leading to improved knowledge and self-management abilities.
Laranjo et al. (39)	Mobile health apps	Showed promise in delivering tailored education and facilitating patient engagement through conversational agents.
Liang et al. (64)	Mobile health apps	Highlighted the widespread use of smartphones in advanced and emerging economies, indicating their potential for health-related purposes.
Richards et al. (65)	Mobile health apps	Showed positive impacts on self-management behaviours and treatment adherence, empowering patients to actively participate in their care.
Hollander et al. (40)	Telemedicine platforms	Enabled real-time access to patient information, facilitating informed decisions and multidisciplinary collaboration, thereby enhancing clinical decision-making.
Eldaly et al. (66)	Telemedicine platforms	Facilitated seamless information sharing, reducing medical errors and enhancing care coordination among healthcare providers.
Sutton et al. (67)	Telemedicine platforms	Described the potential of mobile health apps equipped with decision support systems to enhance clinical decision-making by providing evidence-based recommendations to healthcare providers.
Roh and Won (68)	Online health information resources	Identified concerns regarding the reliability and accuracy of online health information, particularly for individuals with low health literacy.
Yao et al. (69)	Online health information resources	Explored the concept of eHealth literacy and the need to address the digital divide in accessing and evaluating health information online.
Belfrage et al. (70)	Online health information resources	Highlighted the importance of protecting sensitive health data and ensuring transparent data governance to maintain patient trust in digital health platforms.

## Evidence of improved health outcomes

### Patient education and empowerment

Patient education plays a crucial role in improving health outcomes by enhancing individuals' knowledge and understanding of their conditions. Digital communication tools have been shown to effectively educate and empower patients. For instance, a study by Greenwell et al. (51) demonstrated that a mobile app significantly improved patients' knowledge and confidence. Additionally, a systematic review by Laranjo et al. (39) highlighted the potential of conversational agents in healthcare to provide personalised education and support to patients, leading to improved health outcomes.

### Self-Management and treatment options

Digital communication tools offer opportunities for individuals to actively participate in self-management and explore various treatment options. Mobile health apps have been associated with improved self-management behaviours and outcomes in chronic conditions (51, 57). Telemedicine has also demonstrated positive effects on self-care and adherence to treatment plans, as observed in a randomised controlled trial by Buvik et al. (57), where remote orthopaedic consultations resulted in comparable quality of care to in-person consultations.

### Enhanced clinical decision-making

Digital communication tools facilitate data collection, analysis, and communication, leading to enhanced clinical decision-making. Telemedicine consultations provide real-time access to patient information and enable multidisciplinary collaboration, contributing to improved clinical outcomes (40). Additionally, mobile health apps equipped with decision support systems have shown potential in assisting clinicians in making evidence-based decisions, as highlighted in the study by Laranjo et al. (39).

### Improved communication among providers

Effective communication among healthcare providers is vital for coordinated care and improved patient outcomes. Digital communication tools, such as telemedicine platforms, enable seamless communication and information sharing between providers, leading to better care coordination (57, 65). This improved communication facilitates collaboration, reduces medical errors and enhances overall patient care.

## Challenges and limitations of digital health literacy

The utilisation of digital communication tools to improve health literacy has enormous potential but there many challenges and limitations. Recognising the obstacles is crucial for developing effective strategies and interventions.

### Access and connectivity barriers

Access to digital communication tools and reliable internet connectivity remains a significant barrier for certain populations, leading to disparities in digital health literacy. Limited access to smartphones, computers and high-speed internet, often referred to as the digital divide, disproportionately affects underserved communities, older adults and individuals with lower socioeconomic status (43, 62). Addressing the access barriers through initiatives such as community-based technology programs and affordable internet access can help improve digital health literacy across diverse populations.

### Reliability and accuracy of online information

The enormous amount of health information available online presents challenges in terms of reliability and accuracy. Individuals with low health literacy may struggle to discern credible sources from misleading or inaccurate information (62). Lack of discernment can lead to poor decision-making and health outcomes (71, 72). Promoting health literacy skills is crucial to empower individuals to critically evaluate online information and identify reputable sources.

### Privacy and security concerns

Digital communication tools involve the sharing of sensitive personal health information, raising concerns about privacy and security. Safeguarding sensitive health data is paramount to maintain individuals' trust and ensure the ethical use of digital health platforms (70, 71). Relevant regulations, robust data protection measures and transparent data governance are necessary to address privacy and security concerns in the digital health landscape.

### Health inequities and inclusion

Digital health literacy initiatives should address the potential for exacerbating existing health inequities. Vulnerable populations, including those with lower health literacy, limited digital skills or language barriers, may face additional challenges in navigating and utilising digital health resources (61, 72, 73). Ensuring inclusivity and tailoring digital health interventions to diverse populations can help mitigate disparities. Designing user-friendly, culturally appropriate digital tools and providing tailored support can promote equitable access and usage of digital health platforms (20, 24, 47). Social media is also a powerful means to increase and promote health communication strategies and effective data dissemination (59, 60, 74). The use of social media for advocacy and communications in health promotion offers exciting new prospects for broader reach, greater efficiency and reduced costs of communication and advocacy campaigns.

## Digital tools for clinical decision making

Digital tools have revolutionised the landscape of clinical decision making, offering healthcare providers a wide range of resources and technologies to enhance diagnostic accuracy, treatment planning and patient management.

### Decision support systems

Decision support systems (DSS) play a crucial role in providing evidence-based guidance to healthcare providers during the decision-making process. These systems integrate patient-specific data, clinical guidelines and medical knowledge to generate recommendations and assist healthcare professionals in making informed decisions.

Numerous studies have demonstrated the effectiveness of DSS in improving clinical decision making. For instance, Sutton et al. (67) conducted a review of Clinical DSS in healthcare and highlighted their potential to enhance diagnostic accuracy, treatment selection and patient outcomes. The study also highlighted the importance of vigilance for potential drawbacks of Clinical DSS, which range from simply not working and wasting resources, to fatiguing providers and compromising quality of patient care.

### Artificial intelligence

Artificial intelligence (AI) has emerged as a transformative technology in healthcare, offering powerful algorithms and machine learning techniques to analyse vast amounts of data and provide insights for clinical decision making. AI-based tools have demonstrated remarkable capabilities in various domains, including image analysis, natural language processing and predictive modelling (75).

In the context of clinical decision making, AI has shown promising results. Research by Kumar et al. (76) and Zhang et al. (77) examined the use of AI algorithms in cancer and diagnosis. The studies revealed that AI algorithms achieved high accuracy rates in diagnosing cancer subtypes, assisting clinicians in developing tailored treatment plans. Similarly, Esteva et al. (78) demonstrated the potential of AI-powered deep learning models in detecting skin cancer, matching the performance of dermatologists.

### Predictive analytics

Predictive analytics utilises statistical modelling and machine learning techniques to forecast future events and outcomes based on historical data. In the context of clinical decision making, predictive analytics enables healthcare providers to identify patients at risk, optimise treatment plans and allocate resources efficiently.

Several studies have showcased the utility of predictive analytics in healthcare. For instance, Baird et al. (79) explored the use of predictive models to identify patients at risk of readmission. The findings demonstrated that predictive analytics could accurately predict hospital readmissions, allowing healthcare providers to intervene and provide targeted interventions to reduce readmission rates. Additionally, Guo & Chen (80) highlight the potential of predictive analytics in disease surveillance and early detection, enabling proactive and preventive measures.

### Biases in algorithms

Algorithms used in clinical decision making, particularly those powered by machine learning and artificial intelligence, rely heavily on training data sets. Biases present in these data sets can perpetuate disparities and result in biased outcomes. These biases may arise due to various factors, such as underrepresentation or misrepresentation of certain demographic groups in the training data, societal inequalities reflected in the data or systemic biases embedded in the algorithms themselves.

Research by Obermeyer et al. (81) highlighted racial biases in an algorithm commonly used to predict healthcare needs and allocate resources. The study revealed that the algorithm systematically underestimated the healthcare needs of Black patients compared to White patients, leading to potential disparities in access to care. This example underscores the importance of scrutinising algorithms and addressing biases to avoid exacerbating existing healthcare disparities.

### Limited data sets

The use of limited data sets in training algorithms can result in biased outcomes and suboptimal decision making. Limited data sets may lack diversity in terms of demographic representation, geographical locations or specific medical conditions. Consequently, algorithms trained on such data sets may not adequately capture the complexity and variability of patient populations, leading to inaccurate predictions or recommendations.

Research by Wiens et al. (82) examined the performance of machine learning models in predicting mortality rates using electronic health record data. The study found that models trained on data from a single healthcare system had limited generalisability and performed poorly when applied to different healthcare settings or patient populations. This emphasises the need for diverse and representative data sets to ensure robust and unbiased algorithms.

### Addressing biases and limited data sets

Addressing biases in algorithms and overcoming limitations of limited data sets requires concerted efforts from various stakeholders, including researchers, healthcare providers and policymakers. Some strategies to mitigate biases and improve data inclusivity include:

**Enhancing data collection**: Actively seeking diverse and representative data sets that encompass different demographics, geographic regions and medical conditions to ensure inclusivity.
**Transparent algorithm development**: Ensuring transparency in algorithm development, including providing documentation on data sources, model design and evaluation metrics to facilitate scrutiny and identify potential biases.
**Regular audits and evaluations**: Conducting regular audits and evaluations of algorithms to identify biases, disparities and limitations. This process should involve rigorous testing on diverse data sets involving experts from various backgrounds.

Addressing biases in algorithms and overcoming shortfalls of limited data sets is an ongoing endeavour. Future research should focus on developing standardised frameworks for evaluating algorithmic biases and using robust methodologies to address these biases effectively. Additionally, collaborations between researchers, healthcare organisations and policymakers are essential for implementing equitable unbiased digital technologies in clinical decision making.

While digital technologies hold immense potential for enhancing clinical decision making, concerns surrounding biases in algorithms and limited data sets must not be overlooked or underestimated. It is crucial to address these concerns through rigorous evaluation, transparency and inclusive data collection to ensure equitable unbiased healthcare.

## Regulation and governance of digital health tools

The rapid integration of digital technologies in healthcare services delivery and clinical decision making has prompted the need for robust governance and regulation to ensure patient safety, privacy and ethical use of these technologies. As the technology continues to rapidly evolve, striking the necessary balance between governance and innovation presents very difficult considerations.

### Ensuring patient safety and efficacy

As digital technologies become increasingly sophisticated, it is crucial to establish adaptable regulatory frameworks that prioritise patient safety and efficacy. Technologies, such as AI algorithms and medical devices, necessitates a dynamic regulatory environment that can keep pace with advances. Rigorous evaluation processes and standards for testing, validation and monitoring of digital tools are essential to ensure their safety, effectiveness and reliability.

The European Union's Medical Devices Regulation (MDR) implemented in 2017 is an example of an evolving regulatory framework aimed at enhancing patient safety and ensuring the quality of medical devices. The MDR strengthens regulations for digital health products, including software applications and mobile health devices, by introducing stricter requirements for clinical evaluation, post-market surveillance and risk management (83). Similarly, the various guidance documents issued in recent years as part of the FDA's Patient First Drug Development (PFDD) effort in line with the 21st Century Cures Act and the Food and Drug Administration Reauthorization Act 2017 Title 1 (84).

### Privacy and data protection

Healthcare digital technologies generate vast amounts of personal health data, raising concerns about privacy and data protection. Safeguarding patient information is crucial to maintain trust and promote the ethical use of digital technologies. Governance and regulation must address issues related to data privacy, security, consent and data sharing to ensure that patient information is handled appropriately in compliance with privacy requirements.

The General Data Protection Regulation (GDPR) enacted in the European Union in 2018 is a significant step toward protecting individuals' data rights. The GDPR establishes principles and rules for the collection, storage and processing of personal data, including health data. Similar regulations, such as the California Consumer Privacy Act (CCPA) in the United States, aim to protect individuals' privacy rights, enhance transparency and control over personal data (85, 86).

### Ethical considerations

Ethics play a crucial role in the governance and regulation of digital technologies in healthcare. The ethical use of these technologies involves respecting patient autonomy, ensuring fairness, addressing potential biases and unintended consequences. Ethical guidelines and frameworks can assist healthcare providers, researchers and developers in navigating complex ethical considerations.

The Helsinki Declaration by the World Medical Association and ethical guidelines developed by organisations such as the American Medical Association (AMA) and the British Medical Association (BMA) provide overarching ethical principles for healthcare research and practice. These guidelines emphasise the importance of informed consent, privacy, confidentiality, and fairness in the use of digital technologies (87–89).

### AI in clinical decision making

Artificial intelligence (AI) technologies have shown great promise in improving clinical decision making. However, their adoption in healthcare raises important considerations related to governance and regulation. Algorithms trained on insufficient datasets can introduce biases and perpetuate health disparities. It is essential there is appropriate awareness about data set limitations to minimise risk of biases and misinterpretations when using AI in clinical decision making.

Efforts are underway to develop guidelines and standards for the ethical use of AI in healthcare. The American Medical Association (AMA) has outlined principles for the design, implementation and use of AI in healthcare, emphasising transparency, fairness and accountability (88). The European Commission's High-Level Expert Group on Artificial Intelligence has also published ethical guidelines, emphasising human agency, accountability and inclusiveness in AI development and deployment (90).

### Collaboration and international harmonisation

Given the global nature of digital technologies, collaboration and international harmonisation of governance and regulation are crucial. Collaborative efforts among regulatory bodies, policymakers, industry stakeholders and healthcare professionals can facilitate knowledge sharing, harmonise standards and address emerging challenges collectively.

Initiatives such as the Global Digital Health Index, developed by the Global Digital Health Network, aim to assess and track the progress of digital health implementation worldwide. Additionally, international organisations like the World Health Organisation (WHO) work toward developing global guidelines and frameworks for the ethical and responsible use of digital technologies in healthcare (91).

### Adaptability and continual evaluation

Governance and regulation of digital technologies in healthcare must be adaptive to the rapidly evolving technological landscape. Regular evaluation and updates are necessary to address emerging challenges, assess the effectiveness of regulations and incorporate feedback from stakeholders. Continual monitoring and evaluation frameworks can help identify gaps and areas for improvement ensuring that governance and regulation remain effective and relevant.

Oversight of digital technologies in healthcare services delivery and clinical decision making are essential to safeguard patient safety, protect privacy, uphold ethical standards, promote collaboration and adapt to digital advancements. Establishing robust and dynamic governance frameworks, helps ensure the responsible and effective use of digital technologies to enhance healthcare experience for everyone.

The increased utilisation of digital communication tools in healthcare has brought numerous benefits, including improved access to health information, enhanced patient engagement and streamlined communication between healthcare professionals and patients. However, this widespread adoption of digital communication tools has also placed additional burdens on healthcare professionals, particularly in terms of communication volume. Research shows that the rapid influx of messages, notifications and electronic communications can lead to increased workload and time demands for healthcare staff. For instance, a study by Shanafelt et al. (92) reported that physicians spend a significant amount of time on electronic communication, contributing to physician burnout and impacting work-life balance. Furthermore, reviews of the literature conducted by Patel et al. (93), Bakhai et al. (94); McTaggart & Walker (95) highlight that healthcare professionals often struggle to manage the constant influx of digital communication, leading to feelings of overload and a blurring of personal and professional boundaries. It is paramount that healthcare organisations recognise these challenges and implement strategies to mitigate the impact of communication volume on the work-life balance of healthcare staff. This may involve implementing communication guidelines, providing training on effective digital communication practices and developing supportive work environments that encourage appropriate use and boundaries for digital communication. By addressing these challenges, healthcare organisations can harness the benefits of digital communication tools while prioritising the well-being of their healthcare staff.

## Discussion

Digital communication tools have shown great promise in improving health literacy and fostering better health outcomes for patients and practitioners. We have outlined examples that show the power of various digital communication tools, such as mobile health apps, telemedicine and online health information resources, in promoting health and digital literacy. The evidence suggests that these tools have the potential to enhance patient education, self-management, clinical decision-making and provider communication (39, 51, 57).

Patient education and empowerment are critical components of achieving better health outcomes. Mobile health apps have been successful in providing personalised education and support to patients, leading to improved knowledge and confidence in managing their conditions (51, 107). Additionally, conversational agents in healthcare have shown promise in delivering tailored education and facilitating patient engagement (39).

Self-management plays a crucial role in chronic disease management and overall health improvement. Digital communication tools, including mobile health apps and telemedicine, have demonstrated positive effects on self-management behaviours and treatment adherence (51, 57). These tools offer patients access to resources and support, empowering them to take a more active role in their own care (47, 48).

Improved clinical decision-making is another significant benefit of digital communication tools. Telemedicine consultations enable real-time access to patient information, facilitating informed decisions and multidisciplinary collaboration (40). Mobile health apps equipped with decision support systems have the potential to enhance clinical decision-making by providing evidence-based recommendations to healthcare providers (39, 67, 75).

Effective communication among healthcare providers is vital for coordinated care and better patient outcomes. Digital communication tools, such as telemedicine platforms, have been shown to facilitate seamless information sharing, reducing medical errors and enhancing care coordination (51, 57, 64, 66, 108). By enabling efficient communication and collaboration, these tools have the potential to improve healthcare delivery and patient outcomes. Digital tools empower patients by providing access to resources, tracking tools and remote monitoring, enabling them to actively participate in their care. Digital communication tools enable effective collaboration, further empowering healthcare providers to make well-informed decisions and provide timely interventions.

Despite the significant potential of digital communication tools, there are challenges and limitations that need to be addressed. Access and connectivity barriers continue to hinder the equitable use of these tools, particularly among underserved populations (43, 109, 110)^.^ To fully realise the benefit of digital tools in healthcare, efforts must focus on bridging the digital divide and ensuring accessibility for all individuals. It is also important to note that while technology is a very useful tool it cannot compensate for content that is not engaging, easy to consume and appropriate for the audience (73, 111).

The reliability and accuracy of online health information remains a major worry. Individuals with low health literacy may struggle to discern credible sources (8, 9, 74). It is essential to continually promote health literacy skills that empower individuals to critically evaluate online information and seek reputable sources.

Privacy and security concerns are also important considerations in the digital health landscape. Protecting sensitive health data and ensuring transparent data governance are essential to maintain individuals' trust and confidence in using digital health platforms (70, 71). Implementing robust data protection measures, complying with privacy regulations and adopting encryption technologies are vital for safeguarding patient information, see Table 3.

**Table 3:** Some of the key international regulations and guidelines for digital health.

Regulation/Guideline	Brief Description	Reference
EU Medical Device Regulation (MDR)	Establishes regulations for medical devices, including software and apps, sold within the European Union (European Commission, 2017).	European Commission (96)
FDA Digital Health	Provides regulatory oversight for mobile health apps and other digital health technologies in the United States (U.S. Food and Drug Administration, 2023).	U.S. Food and Drug Administration (97)
General Data Protection Regulation (GDPR)	Regulates the processing and protection of personal data for individuals within the European Union (European Commission, 2016).	European Commission (98)
Health Insurance Portability and Accountability Act (HIPAA)	Sets standards for the protection of individuals’ health information and ensures privacy and security in the United States (U.S. Department of Health & Human Services, 2003).	U.S. Department of Health & Human Services (99)
International Medical Device Regulators Forum (IMDRF)	Provides guidance on the regulation of medical devices, including software, to harmonise global regulatory practices (IMDRF, 2023).	IMDRF (100)
ISO 27001:2013	Information technology—Security techniques—Information security management systems—Requirements	International Organisation for Standardization (101)
ISO 13485:2016	Medical devices—Quality management systems—Requirements for regulatory purposes	International Organisation for Standardization (102)
ISO 82304-1: Part 1: 2016 and Part 2: 2021	Health software—Part 1: General requirements for product safety Health software—Part 2: Health and wellness apps—Quality and reliability	International Organisation for Standardization (103)
		International Organisation for Standardization (104)
National Institute for Health and Care Excellence (NICE) Guidelines	Provides evidence-based guidance on the use of digital health technologies, such as mobile apps and telehealth, in the United Kingdom (NICE, 2023).	NICE (105)
World Health Organisation (WHO) Digital Health Guidelines	Offers guidelines on various aspects of digital health, including telehealth, mobile health, and health information systems (WHO, 2023).	World Health Organization (106)

Addressing health inequities and promoting inclusivity in digital health literacy initiatives are also key to achieving better health outcomes. Tailoring interventions to diverse populations and providing support for individuals with varying digital skills, language barriers and lower health literacy levels can help mitigate disparities and ensure equitable access to digital health resources (8, 68, 69). Collaborating with community organisations and stakeholders can help reach underserved populations and ensure equitable access to digital health resources.

Decision support systems, artificial intelligence and predictive analytics, have and will increasingly contribute to clinical decision making. These technologies have shown effectiveness in improving diagnostic accuracy, treatment planning and patient management (51, 57–59, 65). As digital tools continue to advance, it is essential for healthcare providers and researchers to harness their potential and integrate them into clinical practice to enhance patient outcomes and optimise healthcare delivery (53, 56).

In conclusion, digital communication tools offer immense potential to improve health literacy and achieve better health outcomes for patients and practitioners. By promoting patient education and empowerment, supporting self-management and treatment options, enhancing clinical decision-making and improving provider communication, these tools can transform healthcare delivery. However, addressing access barriers, ensuring the reliability of online information, safeguarding privacy and security while promoting inclusivity are critical to maximise the benefits of digital health literacy initiatives.

## References

[1] Centres for Disease Control (CDC). *What is health literacy*. (2023). Available at: https://www.cdc.gov/healthliteracy/learn/index.html (Accessed July 2023).

[2] World Health Organisation (WHO). *Improving health literacy*. (2023). Available at: https://www.who.int/activities/improving-health-literacy (Accessed July 2023).

[3] Shao Y Hu H Liang Y Hong Y Yu Y Liu C Health literacy interventions among patients with chronic diseases: a meta-analysis of randomized controlled trials. Patient Educ Couns. (2023) 114:107829. 10.1016/j.pec.2023.107829 37270933

[4] Yan L Pu C Rastogi S Choudhury R Shekar MK Talukdar G. Evaluating the influence of health literacy and health-promoting COVID-19 protective behaviors on the spread of infection during the COVID-19 pandemic: a meta-analysis. Adv Clin Exp Med. (2023). 10.1007/978-3-031-38176-8. [Epub ahead of print] 37166014

[5] Reddy P Chaudhary K Sharma B Hussein S. Essaying the design, development and validation processes of a new digital literacy scale. Online Inform Rev. (2023) 47(2):371–97. 10.1108/OIR-10-2021-0532

[6] Neter E Brainin E. Association between health literacy, ehealth literacy, and health outcomes among patients with long-term conditions. Eur Psychol. (2019) 24:68–81. 10.1027/1016-9040/a000350

[7] Shahid R Shoker M Chu LM Frehlick R Ward H Pahwa P. Impact of low health literacy on patients’ health outcomes: a multicenter cohort study. BMC Health Serv Res. (2022) 22(1):1–9. 10.1186/s12913-022-08527-9 36096793 PMC9465902

[8] Keene Woods N Ali U Medina M Reyes J Chesser AK. Health literacy, health outcomes and equity: a trend analysis based on a population survey. J Prim Care Community Health. (2023) 14:21501319231156132. 10.1177/21501319231156132 36852725 PMC10071098

[9] Anderson HL Moore JE Millar BC. Comparison of innovative communication approaches in nutrition to promote and improve health literacy. Ulster Med J. (2022) 91(2):85–91. 35722219 PMC9200103

[10] Clifford C. My health, my language-multilingual video messages on health in Ireland. Rural Remote Health. (2023) 23(1):8173. 10.22605/RRH8173 36802706

[11] Kuo CL. The role of nurses in bridging the health literacy gap: empowering patients in the post-pandemic era. Hu Li Za Zhi. (2023) 70(2):4–6. Chinese. 10.6224/JN.202304_70(2).01 36942536

[12] Zaidman EA Scott KM Hahn D Bennett P Caldwell PH. Impact of parental health literacy on the health outcomes of children with chronic disease globally: a systematic review. J Paediatr Child Health. (2023) 59(1):12–31. 10.1111/jpc.16297 36536542

[13] HLS19 Consortium of the WHO Action Network M-POHL. International report of the european health literacy population survey 2019-2021 (HLS19). 2021 (2021). (Accessed July 2023).

[14] Šulinskaitė K Zagurskienė D Blaževičienė A. Patients’ health literacy and health behaviour assessment in primary healthcare: evidence from a cross-sectional survey. BMC Primary Care. (2022) 23(1):223. 10.1186/s12875-022-01809-5 36064351 PMC9446736

[15] Lopez C Kim B Sacks K. (2023). Health literacy in the United States: enhancing assessments and reducing disparities. 10.2139/ssrn.4182046

[16] Australian Bureau of Statistics. Health literacy, Australia 2006. (2006). ABS Publication No, 4233. (Accessed July 2023).

[17] Choudhry FR Ming LC Munawar K Zaidi ST Patel RP Khan TM Health literacy studies conducted in Australia: a scoping review. Int J Environ Res Public Health. (2019) 16(7):1112. 10.3390/ijerph16071112 30925706 PMC6479782

[18] Oliffe JL McCreary DR Black N Flannigan R Goldenberg SL. Canadian Men’s health literacy: a nationally representative study. Health Promot Pract. (2020) 21(6):993–1003. Erratum in: Health Promot Pract. 2019: 1524839919849038. 10.1177/1524839919837625 30884981

[19] Rajah R Hassali MA Murugiah MK. A systematic review of the prevalence of limited health literacy in southeast Asian countries. Public Health. (2019) 167:8–15. 10.1016/j.puhe.2018.09.028 30544041

[20] Faiola A Kamel Boulos MN Bin Naeem S Ur-Rehman A. Integrating social and family support as a measure of health outcomes: validity implications from the integrated model of health literacy. Int J Environ Res Public Health. (2022) 20(1):729. 10.3390/ijerph20010729 36613058 PMC9819503

[21] Nutbeam D. Health education and health promotion revisited. Health Educ J. (2019) 78(6):705–9. 10.1177/0017896918770215

[22] Vaillancourt R Cameron JD. Health literacy for children and families. Br J Clin Pharmacol. (2022) 88(10):4328–36. 10.1111/bcp.14948 34155667

[23] Yamashita T Kunkel SR. An international comparison of the association among literacy, education, and health across the United States, Canada, Switzerland, Italy, Norway, and Bermuda: implications for health disparities. J Health Commun. (2015) 20(4):406–15. 10.1080/10810730.2014.977469 25749096

[24] Sentell T Vamos S Okan O. Interdisciplinary perspectives on health literacy research around the world: more important than ever in a time of COVID-19. Int J Environ Res Public Health. (2020) 17(9):3010. 10.3390/ijerph17093010 32357457 PMC7246523

[25] Nutbeam D Lloyd JE. Understanding and responding to health literacy as a social determinant of health. Annu Rev Public Health. (2021) 42(1):159–73. 10.1146/annurev-publhealth-090419-102529 33035427

[26] Shan Y Ji M Dong Z Xing Z Xu X. Assessing Patients’ critical health literacy and identifying associated factors: cross-sectional study. J Med Internet Res. (2023) 25:e43342. 10.2196/43342 37018027 PMC10132028

[27] National Institutes of Health. *Clear communication clear and simple*. (2021). Available at: https://www.nih.gov/institutes-nih/nih-office-director/office-communications-public-liaison/clear-communication/clear-simple (Accessed July 2023).

[28] Centers for Disease Control and Prevention. *Health equity guiding principles for inclusive communication*. (2022). Available at: https://www.cdc.gov/healthcommunication/Health_Equity.html (Accessed July 2023).

[29] Centers for Disease Control and Prevention. *Your one-stop shop for health communication*. (2023). Available at: https://www.cdc.gov/healthcommunication/index.html (Accessed July 2023).

[30] International Plain Language Federation. *ISO 24495-1:2023 plain language—part 1: governing principles and guidelines*. (2023). Available at: https://www.iso.org/standard/78907.html (Accessed July 2023).

[31] Automated Readability Index (ARI). *Readability assessment tool*. (2023). Available at: https://readabilityformulas.com/automated-readability-index.php (Accessed July 2023). 5302480

[32] Coleman-Liau Index. *Readability assessment tool*. (2023). Available at: https://readabilityformulas.com/coleman-liau-readability-formula.php (Accessed July 2023).

[33] Dale-Chall Readability Formula. *Readability assessment tool*. (2023). Available at: https://readabilityformulas.com/new-dale-chall-readability-formula.php (Accessed July 2023).

[34] Flesch Reading Ease Score. *Readability assessment tool*. (2023). Available at: https://readable.com/readability/flesch-reading-ease-flesch-kincaid-grade-level/ (Accessed July 2023).

[35] FORCAST (Formula for Assessing Readability and Complexity of Text). *Readability assessment tool*. (2023). Available at: https://www.readabilityformulas.com/forcast-readability-formula.php (Accessed July 2023).

[36] Fry Graph Readability Formula. *Readability assessment tool*. (2023). Available at: https://readable.com/readability/fry-readability-graph/ (Accessed July 2023).

[37] Gunning Fog Index. *Readability assessment tool*. (2023). Available at: https://readable.com/readability/gunning-fog-index/ (Accessed July 2023).

[38] Simple Measure of Gobbledygook (SMOG). *Readability assessment tool*. (2023). Available at: https://readable.com/readability/smog-index/ (Accessed July 2023).

[39] Laranjo L Dunn AG Tong HL Kocaballi AB Chen J Bashir R Conversational agents in healthcare: a systematic review. J Am Med Inform Assoc. (2018) 25(9):1248–58. 10.1093/jamia/ocy072 30010941 PMC6118869

[40] Hollander JE Carr BG. Virtually perfect? Telemedicine for COVID-19. N Engl J Med. (2020) 382(18):1679–81. 10.1056/NEJMp2003539 32160451

[41] Sharma S Kar AK Gupta MP Dwivedi YK Janssen M. Digital citizen empowerment: a systematic literature review of theories and development models. Inform Technol Dev. (2022) 28(4):660–87. 10.1080/02681102.2022.2046533

[42] Weitz K Zellner A André E. What do End-users really want? Investigation of human-centered XAI for Mobile health apps. arXiv [preprint]. (2022). Available at: 10.48550/arXiv.2210.03506

[43] Papadopoulos N Cleveland M. An international and cross-cultural perspective on “the wired consumer”: the digital divide and device difference dilemmas. J Bus Res. (2023) 156:113473. ISSN 0148-2963. 10.1016/j.jbusres.2022.113473

[44] International Telecommunication Union (ITU). *Global connectivity report*. (2022). Available at: https://www.itu.int/itu-d/reports/statistics/global-connectivity-report-2022/ (Accessed July 2023).

[45] Digital Cooperation. *Expanding global access to digital infrastructure*. (2022). Available at: https://digitalcooperation.org/internet/ (Accessed July 2023).

[46] Marcolino MS Oliveira JA D’Agostino M Ribeiro AL Alkmim MBM Novillo-Ortiz D. The impact of mHealth interventions: systematic review of systematic reviews. JMIR Mhealth Uhealth. (2018) 6(1):e23. 10.2196/mhealth.8873 29343463 PMC5792697

[47] Bonnechère B Kossi O Mapinduzi J Panda J Rintala A Guidetti S Mobile health solutions: an opportunity for rehabilitation in low-and middle income countries? Front Public Health. (2023) 10:1072322. 10.3389/fpubh.2022.1072322 36761328 PMC9902940

[48] Gayesa RT Ngai FW Xie YJ. The effects of mHealth interventions on improving institutional delivery and uptake of postnatal care services in low-and lower-middle-income countries: a systematic review and meta-analysis. BMC Health Serv Res. (2023) 23(1):1–6. 10.1186/s12913-023-09581-7 37296420 PMC10257264

[49] ORCHA. *Market facts and figures*. (2022). Available at: https://orchahealth.com/market-facts-and-figures/ (Accessed July 2023).

[50] Siberry V Ratwani R Wesley D. Mobile health app privacy policies: what patients want vs. Understand. In: Proceedings of the international symposium on human factors and ergonomics in healthcare. Vol. 11, No. 1. Los Angeles, CA: SAGE Publications (2022). p. 156–61. 10.1177/2327857922111031

[51] Greenwell K Ainsworth B Bruton A Murray E Russell D Thomas M Mixed methods process evaluation of my breathing matters, a digital intervention to support self-management of asthma. NPJ Prim Care Respir Med. (2021) 31(1):35. 10.1038/s41533-021-00248-6 34088903 PMC8178311

[52] ORCHA. *Organisation for the review of care and health apps*. (2023). Available at: https://orchahealth.com/ (Accessed July 2023).

[53] Tan YY Woulfe F Chirambo GB Henn P Cilliers L Fadahunsi KP Framework to assess the quality of mHealth apps: a mixed-method international case study protocol. BMJ Open. (2022) 12(10):e062909. 10.1136/bmjopen-2022-062909 36307160 PMC9621190

[54] National Health Service (NHS) Digital. (2023). Available at: https://digital.nhs.uk/ (Accessed July 2023).

[55] Happtique. (2023). Available at: https://mhealth-hub.org/happtique-medical-app-certification (Accessed July 2023).

[56] Alkhaldi O McMillan B Maddah N Ainsworth J. Interventions aimed at enhancing healthcare Providers’ behavior toward the prescription of mobile health apps: systematic review. JMIR Mhealth Uhealth. (2023) 11(1):e43561. 10.2196/43561 36848202 PMC10012012

[57] Buvik A Bugge E Knutsen G Småbrekke A Wilsgaard T. Quality of care for remote orthopaedic consultations using telemedicine: a randomised controlled trial. BMC Health Serv Res. (2016) 16(1):483. 10.1186/s12913-016-1717-7 27608768 PMC5017045

[58] Lim SL Peng GY Tan MW Sayampanathan AA Tan HC Tay KS. Utility and satisfaction of telemedicine for ambulatory orthopaedic care in an urban setting. Singapore Med J. (2023). 10.4103/singaporemedj.SMJ-2021-333. [Epub ahead of print] PMC13143319 37338489

[59] Li X Liu Q. Social media use, eHealth literacy, disease knowledge, and preventive behaviors in the COVID-19 pandemic: cross-sectional study on Chinese netizens. J Med Internet Res. (2020) 22(10):e19684. 10.2196/19684 33006940 PMC7581310

[60] Niu Z Willoughby J Zhou R. Associations of health literacy, social media use, and self-efficacy with health information–seeking intentions among social media users in China: cross-sectional survey. J Med Internet Res. (2021) 23(2):e19134. 10.2196/19134 33629955 PMC7952238

[61] Patil U Kostareva U Hadley M Manganello JA Okan O Dadaczynski K Health literacy, digital health literacy, and COVID-19 pandemic attitudes and behaviors in US college students: implications for interventions. Int J Environ Res Public Health. (2021) 18(6):3301. ISSN 0148-2963. 10.3390/ijerph18063301 33806763 PMC8004744

[62] Saeed SA Masters RM. Disparities in healthcare and the digital divide. Curr Psychiatry Rep. (2021) 23:61. 10.1007/s11920-021-01274-463. 34297202 PMC8300069

[63] Barello S Triberti S Graffigna G Libreri C Serino S Hibbard J Ehealth for patient engagement: a systematic review. Front Psychol. (2016) 6:2013. 10.3389/fpsyg.2015.02013ISSN=1664-1078 26779108 PMC4705444

[64] Liang X Xiong F Xie F. The effect of smartphones on the self-rated health levels of the elderly. BMC Public Health. (2022) 22(1):1–2. 10.1186/s12889-022-12952-0 35292010 PMC8925210

[65] Richards R Jones R Whittle F Hughes C Hill A Lawlor E Development of a web-based, guided self-help, acceptance and commitment therapy–based intervention for weight loss maintenance: evidence-, theory-, and person-based approach. JMIR Form Res. (2022) 6:e31801. 10.2196/31801 34994698 PMC8783282

[66] Eldaly AS Maniaci MJ Paulson MR Avila FR Torres-Guzman RA Maita K Patient satisfaction with telemedicine in acute care setting: a systematic review. J Clin Transl Res. (2022) 8(6):540–56. 36518201 PMC9741928

[67] Sutton RT Pincock D Baumgart DC Sadowski DC Fedorak RN Kroeker KI. An overview of clinical decision support systems: benefits, risks, and strategies for success. NPJ Digit Med. (2020) 3(1):17. 10.1038/s41746-020-0221-y 32047862 PMC7005290

[68] Roh M Won Y. Impact of online-delivered eHealth literacy intervention on eHealth literacy and health behavior outcomes among female college students during COVID-19. Int J Environ Res Public Health. (2023) 20(3):2044. 10.3390/ijerph20032044 36767409 PMC9915326

[69] Yao R Zhang W Evans R Cao G Rui T Shen L. Inequities in healthcare services caused by the adoption of digital health technologies: scoping review. J Med Internet Res. (2022) 24(3):e34144. 10.2196/34144 35311682 PMC8981004

[70] Belfrage S Helgesson G Lynøe N. Trust and digital privacy in healthcare: a cross-sectional descriptive study of trust and attitudes towards uses of electronic health data among the general public in Sweden. BMC Med Ethics. (2022) 23(1):1–8. 10.1186/s12910-022-00758-z 35246118 PMC8896318

[71] Carini E Villani L Pezzullo AM Gentili A Barbara A Ricciardi W The impact of digital patient portals on health outcomes, system efficiency, and patient attitudes: updated systematic literature review. J Med Internet Res. (2021) 23(9):e26189. 10.2196/26189 34494966 PMC8459217

[72] Cramer A Keinki C Saur F Walter S Hübner J. Ehealth literacy, internet and eHealth service usage: a survey among a German municipality. J Public Health (Bangkok). (2023) 5:1–12. 10.1007/s10389-023-01997-z

[73] Geana MV Anderson S Lipnicky A Wickliffe JL Ramaswamy M. Managing technology, content, and user experience: an mHealth intervention to improve women’s health literacy after incarceration. J Health Care Poor Underserved. (2021) 32(2 Suppl):106–27. 10.1353/hpu.2021.0053 37333690 PMC10275613

[74] Stellefson M Paige SR Chaney BH Chaney JD. Evolving role of social media in health promotion: updated responsibilities for health education specialists. Int J Environ Res Public Health. (2020) 17(4):1153. 10.3390/ijerph17041153 32059561 PMC7068576

[75] Tutun S Johnson ME Ahmed A Albizri A Irgil S Yesilkaya I An AI-based decision support system for predicting mental health disorders. Inform Syst Front. (2023) 25(3):1261–76. 10.1007/s10796-022-10282-5 PMC9142346 35669335

[76] Kumar Y Gupta S Singla R Hu YC. A systematic review of artificial intelligence techniques in cancer prediction and diagnosis. Arch Comput Methods Eng. (2022) 29(4):2043–70. 10.1007/s11831-021-09648-w 34602811 PMC8475374

[77] Zhang B Shi H Wang H. Machine learning and AI in cancer prognosis, prediction, and treatment selection: a critical approach. J Multidiscip Healthc. (2023) 31:1779–91. 10.2147/JMDH.S410301 PMC10312208 37398894

[78] Esteva A Kuprel B Novoa RA Ko J Swetter SM Blau HM Dermatologist-level classification of skin cancer with deep neural networks. Nature. (2017) 542(7639):115–8. 10.1038/nature21056 28117445 PMC8382232

[79] Baird T Eastman L Auger E Moses JM Chand AQ. Reducing readmission risk through whole-person design. NEJM Catal Innov Care Deliv. (2022) 4(1):CAT-22. 10.1056/CAT.22.0237

[80] Guo C Chen J. Big data analytics in healthcare. In: Nakamori Y, editor. Knowledge technology and systems: Toward establishing knowledge systems science. Singapore: Springer Nature Singapore (2023). p. 27–70. 10.1007/978-981-99-1075-5_2

[81] Obermeyer Z Powers B Vogeli C Mullainathan S. Dissecting racial bias in an algorithm used to manage the health of populations. Science. (2019) 366(6464):447–53. 10.1126/science.aax2342 31649194

[82] Wiens J Saria S Sendak M Ghassemi M Liu VX Doshi-Velez F Do no harm: a roadmap for responsible machine learning for healthcare. Nat Med. (2019) 25(9):1337–40. 10.1038/s41591-019-0548-6 31427808

[83] European Commission. *Regulation (EU) 2017/745 of the European parliament and of the council*. (2017). Available at: https://eur-lex.europa.eu/legal-content/EN/TXT/?uri=CELEX%3A32017R0745 (Accessed July 2023).

[84] Food and Drug Administration (FDA). *Development and regulatory decision making 2023*. (2023). Available at: https://www.fda.gov/drugs/development-approval-process-drugs/fda-patient-focused-drug-development-guidance-series-enhancing-incorporation-patients-voice-medical (Accessed July 2023).

[85] European Parliament. *Regulation (EU) 2016/679 of the European parliament and of the council*. (2016). Available at: https://eur-lex.europa.eu/legal-content/EN/TXT/?uri=CELEX%3A32016R0679 (Accessed July 2023).

[86] California Legislative Information. *California consumer privacy act of 2018*. (2018). Available at: https://leginfo.legislature.ca.gov/faces/billTextClient.xhtml?bill_id=201720180AB375 (Accessed July 2023).

[87] World Medical Association. *Declaration of helsinki—ethical principles for medical research involving human subjects*. (2018). Available at: https://www.wma.net/policies-post/wma-declaration-of-helsinki-ethical-principles-for-medical-research-involving-human-subjects/ (Accessed July 2023).

[88] American Medical Association (AMA). *Advancing healthcare AI through ethics, evidence and equity*. (2023). Available at: https://www.ama-assn.org/practice-management/digital/advancing-health-care-ai-through-ethics-evidence-and-equity (Accessed July 2023).

[89] British Medical Association. *2023 ethics*. Available at: https://www.bma.org.uk/advice-and-support/ethics (Accessed July 2023).

[90] European Commission. *Artificial intelligence act*. (2023). Available from: https://www.europarl.europa.eu/RegData/etudes/BRIE/2021/698792/EPRS_BRI(2021)698792_EN.pdf (Accessed July 2023).

[91] World Health Organisation. *Global strategy on digital health 2020–2025*. (2021). Available at: https://www.who.int/docs/default-source/documents/gs4dhdaa2a9f352b0445bafbc79ca799dce4d.pdf (Accessed July 2023).

[92] Shanafelt TD Dyrbye LN Sinsky C Hasan O Satele D Sloan J West CP. Relationship between clerical burden and characteristics of the electronic environment with physician burnout and professional satisfaction. In: Mayo clinic proceedings. Vol. 91, No. 7. Elsevier (2016). p. 836–48. 10.1016/j.mayocp.2016.05.007 27313121

[93] Patel RS Bachu R Adikey A Malik M Shah M. Factors related to physician burnout and its consequences: a review. Behav. Sci. (Basel). (2018) 8(11):98. 10.3390/bs8110098 30366419 PMC6262585

[94] Bakhai A McCauley L Stones L Khalil S Mehta J Price N Shining a light on an additional clinical burden: work-related digital communication survey study–COVID-19 impact on NHS staff wellbeing. Humanit Soc Sci Commun. (2022) 9(1):1. 10.1057/s41599-022-01427-7 PMC9676904 36439048

[95] McTaggart LS Walker JP. The relationship between resident physician burnout and its’ effects on patient care, professionalism, and academic achievement: a review of the literature. Health Sciences Review. (2022):100049. ISSN 2772-6320. 10.1016/j.hsr.2022.100049

[96] European Commission. *Medical devices regulation (MDR) (EU) 2017/745*. (2017). Available at: https://eur-lex.europa.eu/legal-content/EN/TXT/?uri=CELEX%3A32017R0745 (Accessed July 2023).

[97] Federal Drug Administration (FDA). *FDA digital health*. (2023). Available at: https://www.fda.gov/medical-devices/digital-health-center-excellence (Accessed July 2023).

[98] European Commission. *General data protection regulation (GDPR)*. (2016). Available at: https://gdpr-info.eu/ (Accessed July 2023).

[99] U.S. Department of Health & Human Services. *Health insurance portability and accountability act (HIPAA)*. (2003). Available at: https://www.hhs.gov/hipaa/for-professionals/privacy/laws-regulations/index.html (Accessed July 2023). 10.3109/15360288.2015.1037530 26095483

[100] International Medical Device Regulators Forum (IMDRF). (2023). Available at: https://www.imdrf.org/ (Accessed July 2023).

[101] International Organisation for Standardisation. *ISO 27001: 2013 information technology—security techniques—information security management systems—requirements*. (2013). Available at: https://www.iso.org/standard/27001 (Accessed July 2023). 10.1038/160389a0 20263978

[102] International Organisation for Standardisation. *ISO 13485: 2016 medical devices—quality management systems—requirements for regulatory purposes*. (2016). Available at: https://www.iso.org/iso-13485-medical-devices.html (Accessed July 2023). 10.1038/160389a0 20263978

[103] International Organisation for Standardisation. *ISO 82304-1: 2016 health software—part 1: general requirements for product safety*. (2016). Available at: https://www.iso.org/standard/59543.html (Accessed July 2023). 10.1038/160389a0 20263978

[104] International Organisation for Standardisation. *ISO 82304-2: 2021 health software—part 2: health and wellness apps—quality and reliability*. (2016). Available at: https://www.iso.org/standard/78182.html (Accessed July 2023). 10.1038/160389a0 20263978

[105] National Institute for Health and Care Excellence (NICE) Guidelines. *Digital health technologies*. (2023). Available at: https://www.nice.org.uk/about/what-we-do/digital-health/office-for-digital-health (Accessed July 2023).

[106] World Health Organisation (WHO). *Digital health*. (2020). Available at: https://www.who.int/health-topics/digital-health (Accessed July 2023).

[107] Xu L Shi H Shen M Ni Y Zhang X Pang Y The effects of mHealth-based gamification interventions on participation in physical activity: systematic review. JMIR Mhealth Uhealth. (2022) 10(2):e27794. 10.2196/27794 35113034 PMC8855282

[108] Kruse CS Krowski N Rodriguez B Tran L Vela J Brooks M. Telehealth and patient satisfaction: a systematic review and narrative analysis. BMJ Open. (2017) 7(8):e016242. 10.1136/bmjopen-2017-016242 28775188 PMC5629741

[109] Anderson M Perrin A. Tech adoption climbs among older adults. Washington, DC: Pew Research Center (2017). Available at: https://www.pewresearch.org/internet/2017/05/17/tech-adoption-climbs-among-older-adults/

[110] Horwood J Pithara C Lorenc A Kesten JM Murphy M Turner A The experience of conducting collaborative and intensive pragmatic qualitative (CLIP-Q) research to support rapid public health and healthcare innovation. Front Sociol. (2022) 7:970333. 10.3389/fsoc.2022.970333 36189441 PMC9520785

[111] Paige SR Alber JM Stellefson ML Krieger JL. Missing the mark for patient engagement: mHealth literacy strategies and behavior change processes in smoking cessation apps. Patient Educ Couns. (2018) 101(5):951–5. 10.1016/j.pec.2017.11.006 29153592 PMC5911212

